# Prevalence and Risk Factors Associated with *Toxocara canis* Infection in Children

**DOI:** 10.1155/2013/572089

**Published:** 2013-06-09

**Authors:** Camilo Romero Núñez, Germán David Mendoza Martínez, Selene Yañez Arteaga, Martha Ponce Macotela, Patricia Bustamante Montes, Ninfa Ramírez Durán

**Affiliations:** ^1^Centro Universitario Amecameca, Universidad Autónoma del Estado de México, 56900 Amecameca, MEX, Mexico; ^2^Departamento de Producción Agrícola y Animal, Universidad Autónoma Metropolitana, 04960 Mexico City, DF, Mexico; ^3^Parasitología Experimental, Instituto Nacional de Pediatría de México, 04530 Mexico City, DF, Mexico; ^4^Facultad de Medicina, Universidad Autónoma del Estado de México, 50180 Toluca, MEX, Mexico

## Abstract

The objective of this study was to determine seroprevalence and identify risk factors associated with *Toxocara canis* infection. A clinical and epidemiological questionnaire and body mass index were used to assess the risk factors associated with human toxocariasis in 108 children with an age range of 2–16 years. Antibodies against *Toxocara canis* were detected using an ELISA test kit. Chi-square analysis and odds ratio (OR) were used to identify risk factors associated with *Toxocara canis* seropositivity. The prevalence of antibodies against *Toxocara canis* was greater (*P* = 0.02) in males than females (28.84% and 16.07%, resp.). Chi-square analysis and odds ratio revealed just one variable with *P* < 0.05, and OR > 1.0 was associated with seropositivity: the possession of dogs under one year old (OR = 1.78). Although not significant, the OR values suggest that other factors may be epidemiologically important for * Toxocara* presence such as not washing hands before meals, malnutrition, obesity, and use of public parks. Children in the age group >12 and <16 years old had higher seroprevalence to *Toxocara canis* (17.59%) than the >2 and <11 years old age group (4.62%). Toxocariosis infection needs to be prevented by pet deworming and hygienic measures after contact with dogs.

## 1. Introduction

Toxocariasis is a parasitic zoonosis that causes significant morbidity worldwide. It is caused by nematodes of the genus *Toxocara*, which includes more than 30 species, of which two are important for humans: *Toxocara canis* and *Toxocara cati* [1]. Infection occurs in children by ingestion of eggs from *T. canis* via hands contaminated by direct contact with puppies, through the hair of the dog [2], or by ingestion of vegetables or soil contaminated with* Toxocara* eggs [3]. Consumption of raw liver from *Toxocara*-infected paratenic hosts is a less frequent source of contamination [4].

 Seroprevalence in children is variable and can be as low as 1% reported in Spain [5] or as high as 67% reported in Argentina [6]; within each region or country prevalence can also vary widely, as in Brazil where there are locales with 8.7% and others up to 54.8% rates [7, 8]. Geophagia and the use of public parks have been recognized as risk factors for *Toxocara* in children but others have been poorly evaluated [9], so the objective of this study was to evaluate the seroprevalence of *Toxocara canis* in children and its association with several factors such as their health, hygiene practices, and pets.

## 2. Materials and Methods

### 2.1. Study Location

 A cross-sectional study was carried out between August and September 2010 in the Municipality of Ecatepec of Morelos (Latitude 19° 36′03′′N and Longitude 99° 03′09′′W). Ecatepec is a city in the metropolitan area of Mexico City located in the north of the State of Mexico and north of the Mexican Valley; it has a mean altitude of 2,259 m above sea level and is an area with high population density and industrial zones. This district has a population of 1,656,107 inhabitants. Ecatepec has a temperate subhumid climate with rains in summer, with a mean temperature of 13.8°C (between 7 and 30°C) and an average relative humidity of 35%. 

### 2.2. Data Collection

Interviews were carried out in the schools of participants, with informed consent from a parent and/or legal guardian and the assent of the children. A total of 108 subjects from 24 months to 16 years old participated in this research, with parental agreement for this study. This research had the approval of the Ethical Research Committee of the Graduate School of Medicine faculty, Universidad Autónoma del Estado de México.

 Data from the questionnaires included risk factors and epidemiological data (age, sex, dog ownership, dog presence indoors, dogs sleeping with people, older dogs, pet deworming, presence of other pets, use of public parks, history of onychophagia, not washing hands, and eating undercooked meat). The clinical data considered included respiratory signs (bronchospasm, bronchitis, and asthma), dermatologic signs (rash, hives, and itching), and BMI as a measure of nutritional status [10]. Children with BMI less than 20 kg/m^2^ were considered underweight or malnourished, those with BMI greater than 30 kg/m^2^ were categorized as overweight or obese, and the rest were considered as having normal nutrition [11].

### 2.3. Laboratory Analyses

Blood samples were collected for serology and assessment of eosinophils. All sera were analyzed by ELISA at the Parasitology Laboratory of the National Institute of Pediatrics, Mexico City, DF, using a commercial kit with *Toxocara *IgG (SCIMEDX Toxocara IgG). Values of optical density greater than 0.3 according to the serological ELISA test were considered positive. The numbers of circulating eosinophils were evaluated; “eosinophilia” was used to describe those with more than 10% eosinophils or an absolute value greater than 500 cells/*μ*L of IgG [12].

### 2.4. Statistical Analysis

 Analysis was carried out using Chi-square and odds ratio (OR) tests in order to confirm the mean differences between groups and association with seroprevalence [13]. The level of significance selected was *P* < 0.05 with an odds ratio >1.0.

## 3. Results

Twenty-four (22.22%) serum samples from the 108 examined sera were *T. canis* seropositive (Table 1). The prevalence of antibodies against *T. canis* was greater (*P* = 0.02) in males than females (28.84% and 16.07%, resp.). The seroprevalence to *T. canis* tended be lower in the youngest children (4.62%) compared with the oldest group (17.59%) as shown in Table 1.

 Analysis of the signs/symptoms suggested no relationship between respiratory signs or symptoms (20.0%) or cutaneous signs (36.0%) with Toxocariasis, although the odds ratio was greater than 1 in the latter case (Table 2). Eosinophilia was statistically associated with positive serology (Table 2).

 The analysis of risk factors evidenced that malnutrition, obesity, not washing hands before meals, onicophagia, and use of public parks all showed significant associations with seropositivity, although the OR values (>1.0) suggest that they may be epidemiologically important for *Toxocara* presence (Table 3). Nevertheless, the presence of dogs younger than 1 year old within households showed a significant association with seropositivity (Table 3).

## 4. Discussion

The seroprevalence rate found (22.22%) was lower than that reported in children from India (32.86%) [14] and two studies from Argentina which reported 39% and 67%, respectively [6, 15]. In Brazil, it was found that children of 5–8 years old were more likely to be positive for *Toxocara canis* [8], contrary to the findings in this study where the older group had more seropositives. Another study showed a higher prevalence in groups from 1 to 10 years of age, which was related to pica and contact with puppies [16].

 In both age ranges, boys were more prone to be infected with *Toxocara canis *than girls. This has been observed in several studies with different degrees of magnitude. In India, boys had double the infection rate (41.9%) of girls (20.4%) in the 1 to 16 years old age group [14] which was similar to that reported in children in Brazil, with 66.7% prevalence in boys and 33.3% in girls [8]. The eradication of dogs in Iceland during the forties resulted in the disappearance of Toxocariasis in humans by the early eighties [17]. A study in Brazil showed an association between increased levels of *Toxocara* antibodies in children and the presence of young dogs and contact with their feces [7].

 The presence of dogs in the household is considered a risk factor, for the presence of *Toxocara* seropositivity [18]. In this study the presence of dogs younger than one year of age was found to be a significant risk factor, but this association is not always detected [9], perhaps because in studies the age of the dog is not given, and younger dogs are more susceptible to toxocariasis [14]. A higher prevalence of *Toxocara* has also been reported in unfenced houses, indicating that the entry of dogs into the home is also a source of infection for children [14].

 A lack of hand washing before meals was not significantly associated but had an OR of 1.73. It was found that 80% of positive children did not wash their hands before eating, doing nothing to prevent contamination after contact with puppies or with soil or sand contaminated with *Toxocara *eggs [19]. A lack of washing of fruits and vegetables was not associated with serology positive; however some studies indicate that they can be sources of contamination. Only a low percentage (1.5%) of raw vegetables used for salads was found to be contaminated with *Toxocara* spp. [20]. The problem may be larger, as in Libya eggs were found to be contaminated with *Toxocara canis* and *Toxocara cati* in 11% to 48% of 127 samples of raw salads [21]. In Mexico, contamination was demonstrated in carrots and radishes that were not disinfected correctly [22]. The consumption of raw vegetables grown in soils contaminated with *Toxocara* can be a source of chronic infection [23].

 Although there was no association of seropositives with respiratory problems, bronchospasm, shortness of breath, and cough have been reported in children with Toxocariasis [24]. Of the patients with positive serology, 60% had eosinophilia. Among the causes of eosinophilia are parasitic and allergic diseases, with the latter considered to be the main diagnosis [23]. An increase in eosinophils has been associated with *Toxocara* [12].

 Although nutritional status was not associated with toxocariasis serology, the odds ratio values indicate that low weight/malnutrition (OR = 1.46) and overweight/obesity (OR = 1.22) can be considered as potential risk factors. An association between BMI and *Toxocara* has not been reported; however, correlation between the presence of helminthes and nutritional status has been evaluated in Venezuela, in a group of 257 children and adolescents, where intestinal parasites were associated with the presence of malnutrition, with 43.5% of malnourished children parasitized [25]. In a study conducted in Argentina with 248 children, 24.2% had some form of malnutrition (10.9% were malnourished and 13.3% were overweight) and 74.6% of these had parasites, with the 8.9% of children with malnutrition and parasitism representing a slightly higher prevalence in malnourished than in normally nourished children [10].

 There is one study about seroprevalence for *Toxocara *among children along the Mexico-United States border region with 10.6% positives [26]; however, the infestation potential is very high in the metropolitan area in the State of Mexico since the soils in public parks are highly contaminated (30.3%), as well as dog feces found in soil (28.1%) or sampled from dogs with owners (39.8%) [27]. Environmental contamination and the high prevalence of parasitized dogs explain the seropositivity found in this study and should be considered as important aspects for Toxocariasis prevention in children.

## 5. Conclusions 

It is concluded that boys had a greater presence of antibodies than girls, and seroprevalence increased with age. The medium rate for Toxocariasis seroprevalence found in this study in children could be the result of several factors, however, the results indicate that ownership of younger dogs in the home is the main risk factor associated with Toxocariasis. Other factors, including malnutrition, obesity, not washing hands before meals, and use of public parks, were not significantly associated with seropositivity to *Toxocara canis* in this study; however, future studies should be conducted to better assess the importance of these factors in this zoonotic disease.

## Figures and Tables

**Table 1:** Seroprevalence of *Toxocara canis* in boys and girls from 2 to 16 years of age.

Age	Seropositives		*P*-Fisher
	Males (*n* = 52)	Females (*n* = 56)	
2–11 years	3	2	0.31
12–16 years	12	7	0.09

Total	15	9	0.02

**Table 2:** Health problems and nutritional status associated with toxocariasis serology in children from Ecatepec, Mexico.

Problem/nutrition status	Positives (*n* = 24)	Negatives (*n* = 84)	Chi^2^	*p*	OR	(95% CI)	*P*
Respiratory	5	21	0.29	0.58	0.73	0.246–2.212	0.58
Dermatological	9	22	0.84	0.35	1.55	0.602–4.037	0.35
Eosinophilia	15	21	10.4	0.001	4.42	1.728–11.346	0.001
Normal nutrition	12	41	0.01	0.99	0.94	0.386–2.313	0.90
Low weight/mal nutrition	9	23	0.63	0.42	1.46	0.568–3.785	0.42
Overweight/obesity	6	17	0.14	0.70	1.22	0.424–3.543	0.707

*OR: odds ratio; CI: confidence intervals; *p*: probability value for Chi-square; *P*: probability value for OR.

**Table 3:** Some factors evaluated for their association with toxocariasis serology in children from Ecatepec, Mexico.

Factors	Positives (*n* = 25)	Negatives (*n* = 83)	Chi^2^	*p *	OR	(95% CI)	*P *
Not washing hands	10	23	1.36	0.24	1.73	0.683–4.423	0.24
Not washing fruits and vegetables	1	4	—	—	0.82	0.087–7.718	0.86
Consumption of raw meat	4	15	0.057	0.81	0.86	0.258–2.885	0.81
Onicophagia	13	38	0.29	0.58	1.28	0.523–3.141	0.58
Use of public parks and gardens	19	54	1.95	0.30	1.70	0.611–4.729	0.30
Ownership of dogs	18	70	1.93	0.16	0.47	0.166–1.371	0.16
Ownership of dogs younger than 1 year	18	49	1.37	0.24	1.78	0.672–4.737	0.24
Dog is not dewormed	6	17	0.14	0.70	1.22	0.4241–3.548	0.70
Dogs sleep with the owner	1	23	—	—	0.10	0.013–0.850	0.03
Frequent contact with dogs	11	45	0.80	0.37	0.66	0.269–1.632	0.37

*OR: odds ratio; CI: confidence intervals; *p*: probability value for Chi-square; *P*: probability value for OR.
